# Responsibility with a Safety Net: Exploring the Medical Student to Junior Doctor Transition During COVID-19

**DOI:** 10.1007/s40670-021-01476-8

**Published:** 2021-12-01

**Authors:** Chris Wilkinson, Gabrielle Finn, Paul Crampton

**Affiliations:** 1 Faculty of Medical Sciences, Population Health Sciences Institute, Newcastle University, William Leech Building, Newcastle upon Tyne, NE2 4HH UK; 2 Health Professions Education Unit, Hull York Medical School, York, UK; 3 Faculty of Biology, Medicine and Health, University of Manchester, Manchester, UK

**Keywords:** Preparedness, Transitions, Foundation, COVID-19, Interim, Qualitative, Phenomenology

## Abstract

**Introduction:**

The Foundation Interim Year-one (FiY1) Programme was part of a UK strategy to increase the medical workforce in response to the COVID-19 pandemic. However, the strategy was introduced urgently without evidence. We sought to explore the transition experience of medical student to FiY1 to foundation doctor, with a view to inform future undergraduate education.

**Methods:**

In this hermeneutic phenomenology study, semi-structured individual interviews were completed with nine foundation doctors who had experience of an FiY1 placement. A template analysis approach was taken, and themes reported.

**Results:**

Participants reported that FiY1 tended to offer a positive experience of transition as a stepping stone to becoming a foundation doctor. Having a degree of clinical responsibility including the right to prescribe medication with supervision was highly valued, as was feeling a core member of the healthcare team. Participants perceived that FiY1 made them more prepared for the foundation transition, and more resilient to the challenges they faced during their first foundation job.

**Discussion:**

The FiY1 fostered many opportunities for junior doctors to bridge the transition to foundation doctor. Aspects of the FiY1 programme, such as early licencing and increased team membership, should be considered for final-year students in the future.

## Introduction

There are key transition periods in the career of a medical student—most notably from high school to university, from pre-clinical to clinical training, and upon starting work as a doctor [1–6]. Historically, junior doctors have reported feeling underprepared for starting work [7, 8], which may reflect factors including the following: personal characteristics; undergraduate training experiences; and familiarity with the task, context, and team [9, 10]. There is a large component of self-efficacy in addition [11], whereby preparedness encompasses aspects of perceived performance, competence, confidence, and nervousness—as well as a judgement on whether the learner feels that they have learned the right things for the task they are being asked to undertake [12], as preparedness is considered to be highly task-specific [13].

**Fig. 1:**
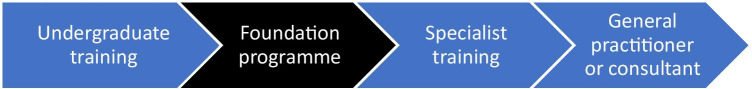
The usual path of medical training in the UK

In the United Kingdom (UK), undergraduate medical degrees are typically of 5-year duration, after which newly qualified doctors are competitively appointed to a 2-year postgraduate training post known as the foundation programme (Fig. 1). During this time, foundation doctors provide direct patient care with the support and supervision of more senior doctors, and receive training in generic skills prior to undertaking specialist training [14]. However, over recent years, there is evidence of an alarming decline in the proportion of FY1 doctors that felt adequately prepared for their first post and identified as a qualified doctor [15]. As a response, a recent review recommended that transition to and preparation for foundation training must urgently be improved [16]. An increased provision of ‘on the job’ training has been identified as a key factor in improving preparedness for starting work, which may include a move towards an apprenticeship model, in which senior medical students often have more engagement and responsibility in clinical environments [2, 17, 18].

In Italy, the USA, and the UK, medical students were brought into the workforce early as part of the national responses to COVID-19 [19, 20]. In the UK, around 80% of final-year medical students were granted early provisional registration by the General Medical Council during the first wave of the pandemic [21]. These graduates with early registration were then eligible to apply for an optional foundation interim year one (FiY1) post through the UK Foundation Programme Office. These roles provided both an accelerated transition into the workplace and the opportunity for supervised clinical work before starting FY1. The posts typically started in April or May 2020 and lasted until August. Those that did not take up an FiY1 post joined the foundation programme as normal in August 2020.

Whilst the circumstances were exceptional across the world, the experiences of FiY1 doctors offer valuable insights at a time of uncertainty, and the opportunity to consider final-year medical student training. The conceptual framework for our study is informed by the preparedness literature, [7, 8] the experiences of on the job learning [13] as well as personal and professional identities [22]. The integrative nature of how these concepts were impacted by COVID-19 may help to extend understanding with a view to better preparing medical graduates to become junior doctors.

In this study, we therefore sought to explore the experience of FiY1 doctors in this unprecedented situation, but also to develop broader understanding on how final-year medical students may be better prepared for the transition between medical student and junior doctor.

## Materials and Methods

Ethical approval was granted by the Hull York Medical School (reference 20/29).

### Study Design

We performed a qualitative study to elicit and understand the experiences of FiY1 doctors. Our intention was to develop a rich understanding of the lived experience of the FiY1 doctors during this unsettling time within the health service. A methodological approach was needed that could delve deep into how participants felt and experienced their posts. Therefore, phenomenology methodology was chosen, which aims to understand a social phenomenon from the perspective of those who have experienced it [23]. More specifically, we chose a hermeneutic phenomenology approach, which acknowledges the importance of the context of participants’ experiences, in this case the clinical environment and wider healthcare context, in order to gain an understanding of those experiences [24]. This approach also recognises that making meaning and constructing knowledge are a collaborative endeavour within the interview, and during analysis [25].

### Participants and Data Collection

Participants were recruited through emails circulated by the administration team at two district general hospitals within a National Health Service (NHS) Foundation Trust in England. Current foundation doctors within the Trust who had completed an FiY1 placement at any site were eligible for inclusion. Potential participants were provided with an information leaflet, and written consent was obtained from those who took part. All those that responded to the invitation met the inclusion criteria and proceeded to interview.

One-to-one semi-structured interviews were completed in September–October 2020. A topic guide was used (Table 1), with questions focused around participants’ experiences to date, preparedness, motivation for taking up an interim placement, experiences since becoming a doctor, and suggestions for future transition improvements. To comply with social distancing measures and decrease the risk to participants and researcher, Zoom video-conferencing software was used. Interviews were audio recorded, and then transcribed verbatim by the researchers.

**Table 1:** Topic guide and example questions

Topic	Example questions
Experiences to date	Did you go straight from school into medical school and then interim? Where did you go to medical school / do your interim placement?
Preparedness	How did you feel about becoming a doctor early? Did you get a chance to complete finals? How did you feel about that? Did you feel ready for the job that you started as an interim doctor?
Motivation for taking up an interim placement	What led to you making your decision to take up an interim placement? How did you feel about becoming a doctor in the middle of a pandemic?
Experiences since becoming a doctor	Do you think that completing the interim placement was helpful in preparing you for being an FY1? Please expand Did you feel ready for becoming an FY1? Do you know people that didn’t do an interim placement? How have they found starting as an FY1?
Learning for the future	Is there anything that you have identified in your experience that we could learn to improve final year for students in the future?

Limited demographic data were collected to preserve anonymity. We report sex, whether participants held a previous degree at the start of their medical degree, whether they had experienced paid employment before, and whether the FiY1 placement was at the same site as their subsequent FY1 job.

### Data Analysis

Through the process of interviewing, contemporaneous notes, verbatim transcription, error checking, and re-reading, the researchers gained familiarity with data. A template analysis approach was then used, whereby a small number of codes were tentatively defined a priori, which were subsequently refined or discarded and a template developed following deep reading and detailed coding of the first two interviews [26]. NVivo 12 (QSR International) was used to record coding decisions. Codes were categorised into themes, which were then narratively summarised with exemplar quotations.

### Bracketing

Within qualitative research, the role of the researcher in constructing meaning is a core part of the study, often referred to as reflexivity [23]. Moreover, within phenomenology, this is commonly termed ‘bracketing’, which is where researchers acknowledge and set aside assumptions about the phenomenon of interest [24, 27]. We discussed and acknowledged that our personal experiences of transitions, uncertainty, and fear surrounding the COVID-19 pandemic and our unique and personal life journeys influence our interpretation of new information [1]. Our professional backgrounds are also likely to influence our interpretation of the data. In particular, the researchers are experienced in postgraduate medical education, as a doctor and teacher (CW) and medical educationalists (PC and GF). CW has experience of transition from medical student to doctor and contributed to the inpatient care of patients with COVID-19. The researchers do not currently work clinically in the Trust and are not involved in teaching, training, or supervising the interviewees so no power relationship exists.

## Results

### Participants

Nine current foundation doctors who had taken part in an FiY1 post were interviewed; their characteristics are detailed in Table 2. Participants were graduates of eight different medical schools and had completed FiY1 placements in seven hospitals across six NHS Hospital Trusts. Four participants continued from their FiY1 placement into FY1 within the same Trust. Interview duration ranged from 20 to 39 (median 32) min.

**Table 2:** Participant characteristics

	**Number (proportion)**
**Sex**	
Women	5 (56%)
Men	4 (44%)
**Qualifications at the start of medical school**	
Undergraduate	7 (78%)
Postgraduate	2 (22%)
**Intercalated degree during medical school**	
Yes	3 (33%)
No	6 (66%)
**Paid employment before FiY1**	
Yes	7 (78%)
No	2 (22%)
**FiY1 placement at the same site as subsequent FY1 job**	
Yes	4 (44%)
No	5 (56%)

### Overview

Key themes identified were in perceived preparedness and transition; a comparison between FiY1 and a standard final-year undergraduate experience; and the relationship between FiY1 and developing resilience. Finally, we present a synthesis of the recommendations and possible opportunities for improving the delivery of final year to better prepare future FY1s, as suggested by the participants.

### Preparedness and Transition

All of the participants recognised that the FiY1 placement had value—and many expressed the view that it had contributed greatly to their feelings of preparedness for the work of FY1:

the eight-week interim period was probably the most beneficial portion of the entire five years of med school for me - and lots of other people that I’ve spoken to would agree with that as well. Dr Seven.

Others felt that fulfilling the role of FiY1 was less daunting than going straight into FY1, which helped with their transition into the doctor role. Whilst the journey from medical student to doctor still entailed transitions, the FiY1 placement ‘smoothed the path’:

I think because it had the name “interim”, that was a nice safety cushion. I didn’t feel like the whole of FY1 responsibility. Dr Four

I felt like I was halfway between being a medical student and being an FY1. I wasn’t entirely like FY1 - FY1 was still a big jump when I started, but it was really useful to have done it, I would say. Dr Five

Preparedness was felt to contribute to a capacity to manage anxiety, and having ready access to FY1s during FiY1 provided additional reassurance:

Definitely I feel, I feel so much more prepared, but also just comfortable, going in and not having a high rate, heart rate sky high. But just being like: “okay it’s high and I know, but I know what’s coming. I know what could be thrown at me and I'm a bit more prepared for that”. Dr Four

it was really good, because there was so many people around to ask and, like, talk me through it, step by step… If I didn’t feel ready I could shadow someone else doing it. Dr Six

This capacity for supervised practice was highly valued by participants, one of whom asked a senior to observe her having a resuscitation discussion for the first time:

I said, “I’ll do it, but can you give me some feedback?”, and having someone to give me that feedback was really useful, and it also meant that if I kind of felt a bit out of depth when I was doing it, or if they asked me a question and I wasn’t too sure, they could step in and support me - which they ended up doing, which was really useful. Dr Six

But there was a balance to be struck—other participants valued having some distance from senior support:

I did feel much more prepared than I think some of my colleagues who were doing interim jobs on medicine did because they were very supernumerary in the sense that a lot of the jobs were so very, very heavily supervised that if there was any even little questions they’d be able to get advice almost straight away. Dr One

The development of institutional knowledge during FiY1 was seen as useful, and therefore, undertaking the role in the same organisation as a subsequent FY1 post would be ideal:

the big thing is IT systems… I wouldn’t even say it’s half the battle. It’s like three quarters of the battle is trying to work out how the computer works! Dr Nine

it’s a lot more useful to be in the same hospital. Obviously the patients are, you know, similar everywhere, but I think being in the same hospital would be the most useful part of it… a lot of the stress of FY1 isn’t actually the situations like the clinical scenarios you have to deal with – it’s actually just the physical logging on the computers and ordering things - and everything takes you just, you know, ten times as long as it needs to, so you can kind of get used to that before you have all the responsibility on top of you as well, then I think it just helps the transition. Dr Five

### Comparison to Being a Final-Year Medical Student

The differences to a standard final-year student experience were of key interest. One participant summarised her experiences:

‘I think, going from “oh I’m just a medical student” and shimmying out of situations to being responsible, people asking your opinion, calling you doctor and you’re just like, “Wow…”! I mean, it wasn’t a learning curve. It’s like a learning straight line!’. Dr Eight

The feeling of personal responsibility and culpability was commonly reported and, for many, was felt to be a driver for learning and integral to the FiY1 experience:

Until… you’re actually doing the job - being there, putting your name on the prescription pad, and thoroughly looking into what the drug does, how it interacts, and what sort of procedures that patient’s gonna have, you’re not really going to click into it…. people don’t really give that much focus unless they are going to be responsible for something... it’s like being in the car, no one who’s sitting in the passenger seat is really fussed about the drive as long as they get from A to B. But the driver is consistently having to look out, adjust accordingly and make all the decisions on which direction to go. And that’s what I felt very much what the interim position was - you were no longer a medical student… you were actually calling the shots. Dr Seven

The key thing is the responsibility. Erm, because if your name’s not on it, if you’re not signing something off, if you’re not actually doing the ward round then you don’t really pay that much attention. It’s sort of padded responsibility, isn’t it? It’s, it’s responsibility with a safety net, which is quite nice bridge. Dr Two

Exposure to the same clinical environment for a sustained period of time was thought to be important for learning during FiY1:

You’re there from eight until five most, most working days. And you see the ins and outs because you are there and people are calling you up and saying, “can you do this, what do you think of this” and you’re, the buck stops with you, essentially. So it really does open your eyes, and preps you for the real situation in August. Dr Seven

This was in contrast to participants’ previous experiences in final year, when they were not always made to feel welcome as learners in the clinical environment. However, working as an FiY1 was a paid role, which made the wards a place of work where attendance was obligatory:

I went on the wards loads, and to the point where it was like “the medical student’s back again, what are we going to do with him?”! And I was just being told to go home all the time, but I was like… “I want to see what's happening. I want to go to theatre, I want to get involved with some of the procedures, and all that sort of stuff because I'm going to be doing it in a year…” Dr Nine

the fact we were paid to be that meant that people couldn’t, like, just send you home or just say, “oh, there’s nothing to do here”. So you were like assigned to that ward and you were paid to be there. So you actually got to do things. We were no more qualified than a fifth [final] year medical student, but there was just that element that they couldn't just tell us to go away as easily! Dr Five

### Resilience

Participants frequently reported that completing an interim placement had enhanced their abilities to recover from adversity and deal with stress—although the magnitude of the perceived benefit was variable:

…the stresses of on calls, weekends, nights, when you’re kind of seeing patients who are acutely unwell. Erm initially, often on your own before somebody else kind of comes in. I would say I feel a little bit more resilient. Dr One

One interviewee had been involved in a critical incident on the day of the interview, and reflected upon whether having completed the interim placement had assisted him in his capacity to, in his words: ‘carry on or bounce back’, but his conclusion suggests that starting work earlier (and therefore having accrued more experience by the time of the interview) was responsible for an improved ability to focus on the big issues, and hence improve resilience, rather than the FiY1 placement itself being a key component:

I feel I took a real shot today, and you know, I got everything that I needed to do done, so… But I can’t say that, like, in general, I’m more or less resilient because of that [the FiY1 placement]. I suppose I knew not to sweat the small stuff a bit better, because I’d had three months of sweating it. Dr Nine

This sense that FiY1 brought forward, rather than mitigated, the stresses of starting work and capacity to build resilience was also experienced by others:

I think it took me quite a while to build up my resilience, because at first I was very easily stressed out, like if things got fast paced or, if like there was too much to do and it was busy, or if I wasn’t really sure what to do. So I think it definitely helped to build up that resilience. Dr Six

### The FiY1 Experience: Possible Lessons for the Future

Participants were asked about whether there were any improvements to training in medical school that they had identified in light of their unanticipated experiences, to better prepare future doctors for the role of FY1. The suggestions that were raised relate to three key themes, which are presented with exemplar quotations: 

1. A feeling of responsibility contributes to effective learning

A distinction was drawn between the experiences gained by a final year medical student during an apprenticeship module, and that of an FiY1. Participants expressed a desire to take on more responsibility for clinical care, and in particular, the routine tasks that they will be expected to perform as an FY1. The current lack of being able to be an active participant in patient care was considered to be demotivating:

‘okay you're doing the apprenticeship, you're being treated like an FY1, but you're not allowed to put your name on the prescription pad. You're not allowed to order the scans. You're not allowed to do X, Y and Z. And I think that just disillusions people from doing those apprenticeships in the first place.’ Dr Seven

2. Students desire to feel a part of the team

FiY1 was often the longest clinical placement in participants’ careers to date, and the first where they felt that they had an important contribution to make – and where they felt part of the team. This sense of belonging was felt to be very valuable:

‘I think people undervalue how important it is for people to feel part of the team. I think as an interim FY1 because I was given a, a role, and it was a doctory role rather than just a student, I felt valued and I felt like I could speak up if I had an opinion, or I had learned something… it helped them out and it helped me with my learning.’ Dr Four

3. The importance ascribed to clinical placements by seniors is influential

Some participants felt that the importance of full attendance on clinical placements was downplayed by clinicians:

‘people just treat it as placement and some, some consultants or clinicians will be very, very lax about it and they'll say, "oh, don't worry about it, you've got you've got the rest of your life to do it, just go off on holiday" and others will be like, "no, you have to do this"’ Dr Seven

Doctors should be aware that this may have a negative impact on the motivation of students on clinical placements to attend and actively engage, and risks making them feel unwelcome.

## Discussion

**Fig. 2:**
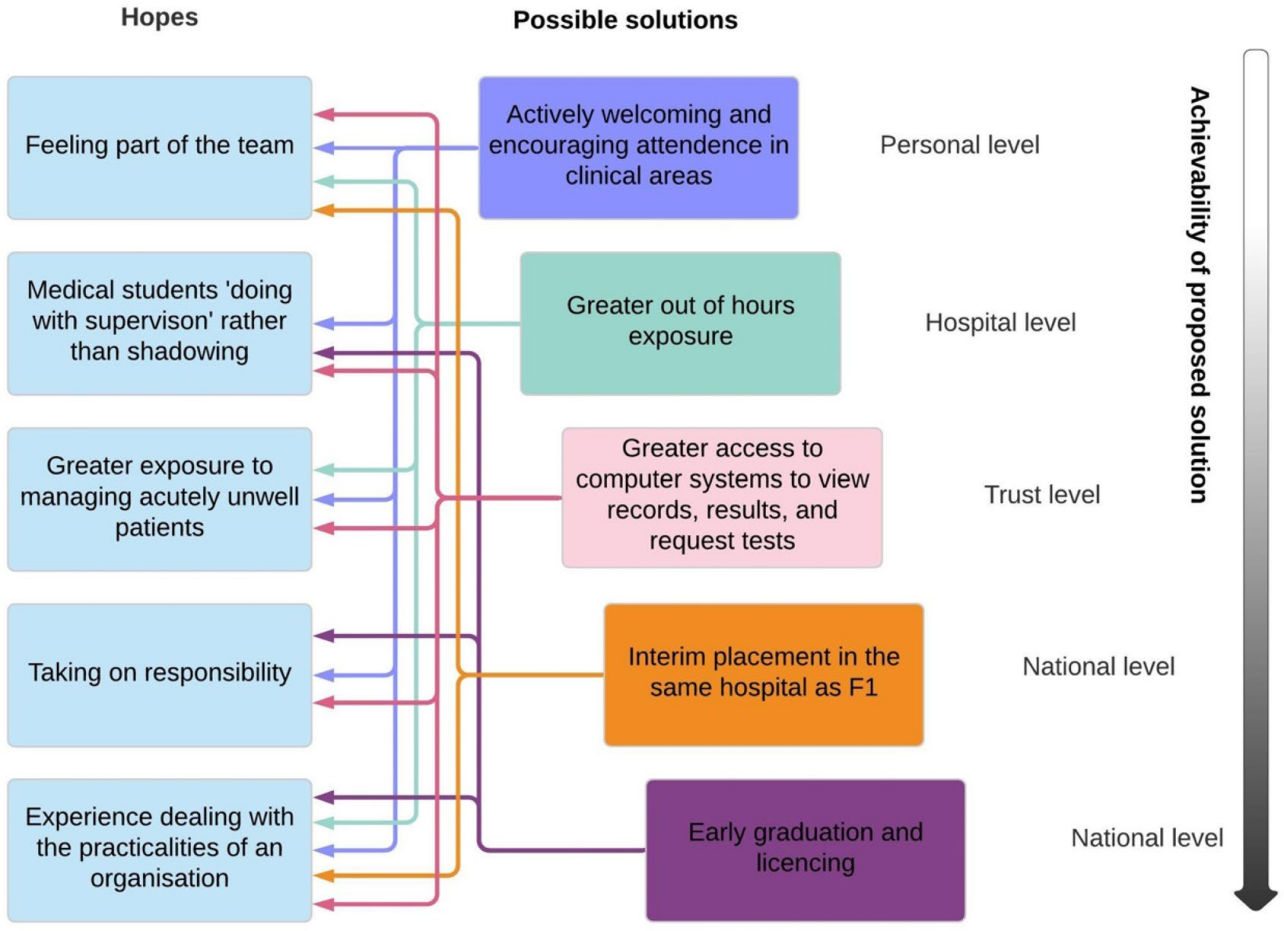
Suggestions on how final-year undergraduate training may be improved. These hopes and possible solutions are based upon the thoughts shared by participants, alongside the views of the authors

To our knowledge, this is the first study of its kind, in which we have identified and explored aspects of the FiY1 programme that added value to the transition between medical student and junior doctor. The experience described by most participants was of being supervised, in situ, and supernumerary—and they felt that the graded transition that this allowed was beneficial in terms of preparedness and resilience. We present a summary of suggestions by the participants’ hopes for improvements in the final-year experience that may better prepare doctors of the future, with suggestions based upon the views of participants and the authors in how these may be achieved (Fig. 2). The authors have also identified at what level change may be achievable, from that achievable by an individual clinician to those that would require action at a national policy level. We believe that the transition findings are of relevance across the world as there were a range of responses by medical authorities in relation to the COVID-19 pandemic.

Despite a challenging backdrop, universities, the General Medical Council, the UK Foundation Office, and the NHS successfully introduced the FiY1 post. The participants were unanimous in considering this to be positive, offering benefits above and beyond an apprenticeship placement. They attributed the added value mostly to being able to actively participate in care (make decision, order investigations, prescribe medications), which has been shown to have a positive effect on perceived learning [28]. Additionally, they also prized having a value and a role within the healthcare team—which may be improved through an increased apprenticeship role for final-year medical students, an approach advocated by the Royal College of Physicians among others [18]. These factors have previously been identified as being of key importance for final-year medical students [2] and, in the case of FiY1, were facilitated by moving provisional registration forwards.

Implementation of this model would enable a supernumerary FiY1-like experience for all new UK graduates alongside current FY1s with the team they will start with in August. This study suggests that there would be benefits for the new doctors in their experience of the transition from medical school—and there are also potential benefits in terms of patient safety and organisational effectiveness. We recognise that this would require widescale change across multiple government and non-governmental organisations. In the meantime, an increased focus on helping medical students feel part of the team and giving them supervised responsibility within the existing framework should be encouraged, although the scope of this will vary by setting, and contingent upon sufficient support and clinical supervision being available to ensure safety and reduce student anxiety.

There may be patient safety and health economic benefits to an interim-like period in non-pandemic times. Medical staffing tends to be subject to cohort turnover [29], despite evidence that there may be differences in mortality and length of hospital stay associated with changeover periods [30, 31]. This is not restricted to newly graduated doctors: excess risk of adverse events has been identified for all levels of medical trainee at the start of a new rotation, which disappears after the fourth month in the job [32]. This suggests that factors beyond clinical competence are likely to be implicated, such as unfamiliarity with the working environment. However, doctors at the very start of their career have limited clinical experience and have not yet had the opportunity to develop in situ communication and teamwork skills, hone clinical acumen, and develop institutional knowledge—factors that may offer a safety net from avoidable harm [33]. An FiY1 role leading into FY1 would provide such an opportunity.

Whilst this study concerned a small number of the clinical workforce, we recognise that COVID-19-related disruption was widespread and affected every sector. A survey of biomedical science PhD students in the USA revealed that one-third experienced a negative impact on their psychological health, with concerns over time management cited as a core contributor to stress [34]. As many remote working practices are likely to persist in future, there is a need for detailed evaluation and consideration of the impact on students across all disciplines to ensure that sufficient supervision and support are available to learners.

This study uses a robust qualitative framework and provides novel and relevant findings. The interviews were conducted within a few weeks of completing the FiY1 placement, which limits bias relating to recall and subsequent experiences. However, we recognise the limitations of our work. The study took place in an unprecedented set of circumstances. As many as one in four doctors were off work either sick or in isolation [35]. Whilst little was known about COVID-19, the potential for long-term consequences was increasingly being recognised including chronic physical ill-health [36], alongside the anxiety, depression, and post-traumatic stress disorder that often follow critical illness [37]. More experienced healthcare workers may not have had capacity to provide as intensive pastoral support as would have been desirable due to pandemic-related personal, clinical, and organisational pressures [38–40]. However, whilst the circumstances were unique, the lessons that we identify are eminently transferrable. If viewed from a positivist objective epistemology [41], we recognise that this study reports the experiences of a small cohort across a single NHS Trust; therefore, we await the results of the ongoing comprehensive evaluation of the FiY1 programme with interest [42]. However, from a subjective ontology, phenomenological studies tend to include smaller samples than other qualitative approaches due to the in-depth positioning of analysis and deeper exploration of participants’ lived experiences [43].

## Conclusion

The FiY1 placement was a novel attempt to expand the workforce in a time of unprecedented challenge. In this study, we explored the experiences of these FiY1 doctors. We found that the interim placement offered the opportunity for new graduates to practice medicine with an increased level of support compared with FY1, which was considered beneficial in terms of preparedness, transition, and resilience. The FiY1 doctors valued being a more active member of the team and having ownership over patient care and decision-making than they usually would in their final year of medical school—whilst still having the ‘safety net’ of a more experienced doctor to call upon. Many of these positive experiences should be considered for integration into undergraduate medical training, which may enhance student’s learning and wellbeing.

## Data Availability

The datasets generated during the current study are not publicly available due to the risk of identifying subjects.
